# Phosphorylation of spleen tyrosine kinase at Y346 negatively regulates ITAM-mediated signaling and function in platelets

**DOI:** 10.1016/j.jbc.2023.104865

**Published:** 2023-06-01

**Authors:** Carol A. Dangelmaier, Margaret Patchin, Dhruv N. Vajipayajula, Hymavathi Reddy Vari, Pankaj K. Singh, Monica N. Wright, John C. Kostyak, Alexander Y. Tsygankov, Satya P. Kunapuli

**Affiliations:** Department of Cardiovascular Sciences, Sol Sherry Thrombosis Research Center, Lewis Katz School of Medicine, Temple University, Philadelphia, Pennsylvania, USA

**Keywords:** platelet, hemostasis, tyrosine, thrombosis, spleen tyrosine kinase

## Abstract

Spleen tyrosine kinase (Syk) is expressed in a variety of hemopoietic cells. Upon phosphorylation of the platelet immunoreceptor–based activation motif of the glycoprotein VI (GPVI)/Fc receptor gamma chain collagen receptor, both the tyrosine phosphorylation and activity of Syk are increased leading to downstream signaling events. Although it has been established that the activity of Syk is regulated by tyrosine phosphorylation, the specific roles of individual phosphorylation sites remain to be elucidated. We observed that Syk Y346 in mouse platelets was still phosphorylated when GPVI-induced Syk activity was inhibited. We then generated Syk Y346F mice and analyzed the effect this mutation exerts on platelet responses. Syk Y346F mice bred normally, and their blood cell count was unaltered. We did observe potentiation of GPVI-induced platelet aggregation and ATP secretion as well as increased phosphorylation of other tyrosines on Syk in the Syk Y346F mouse platelets when compared to WT littermates. This phenotype was specific for GPVI-dependent activation, since it was not seen when AYPGKF, a PAR4 agonist, or 2-MeSADP, a purinergic receptor agonist, was used to activate platelets. Despite a clear effect of Syk Y346F on GPVI-mediated signaling and cellular responses, there was no effect of this mutation on hemostasis as measured by tail-bleeding times, although the time to thrombus formation determined using the ferric chloride injury model was reduced. Thus, our results indicate a significant effect of Syk Y346F on platelet activation and responses *in vitro* and reveal its complex nature manifesting itself by the diversified translation of platelet activation into physiological responses.

Signaling is initiated in many immune cells through clustering of immune receptors. Once clustered, their cytoplasmic tails are phosphorylated on tyrosines in a stretch of amino acids known as an immunoreceptor tyrosine activation motif (ITAM) containing two YXX(L/I) sequences (1, 2). Receptors that bear the ITAM motif include the T- and B-cell antigen receptors on T and B lymphocytes, Fc receptor subtypes on multiple types of blood cells, and the glycoprotein VI (GPVI) receptor on platelets (1, 2). There is also a different group of receptors that contain only one YXX(L/I) motif in the cytoplasmic domain that is known as hemITAM (1, 2). The hemITAM-containing receptors include the C-type lectin-like receptor-2 (CLEC-2) on platelets and immune cells as well as dectin in macrophages (1, 2). Although none of the components of the ITAM or hemITAM are intrinsically enzymatic, their phosphorylation triggers various signaling pathways through recruitment and activation of cytoplasmic protein kinases (3, 4, 5, 6, 7, 8, 9, 10, 11, 12). Spleen tyrosine kinase (Syk) is a critical player in this signaling cascade in multiple cell types (6, 8, 10, 11, 12). The importance of this kinase has been demonstrated in Syk KO mice as the lack of Syk is perinatally lethal (13, 14).

The 72-kDa Syk protein is made up of the N terminally located tandem SH2 domains separated by the interdomain A and a kinase domain located in the C-terminal half. The tandem SH2 domains and the kinase domains are separated by the interdomain B. Syk recruitment to the activated receptor involves the binding of its tandem SH2 domains to the phosphorylated ITAM (pITAM) or hemITAM. Syk contains numerous phosphorylation sites which regulate both its activity and provide a docking site for other proteins. Syk binding to pITAM triggers a downstream signaling cascade (reviewed in (15, 16, 17, 18, 19)). While it has been known that binding of Syk to pITAM leads to Syk phosphorylation at several sites thus increasing its activity, many questions remain regarding the mechanisms leading to this activation. Platelets express the ITAM-containing receptor GPVI as well as the hemITAM receptor CLEC-2, and Syk plays a key role in platelet signaling and activation (20, 21, 22, 23, 24). We chose these cells as a model system to evaluate the role of tyrosines on Syk in its activation.

Platelets are anucleate cells that are produced by megakaryocytes (25). Their major function is to respond to vascular damage and mediate hemostasis (26, 27). Platelets express many cell surface receptors that are necessary for these processes (26, 27). Signals originating from cell surface receptors result in platelet shape change, secretion of granular contents, and production of thromboxane A2, which reinforces the original signal and recruits other platelets. Elucidating how these cell surface receptors signal intracellularly is crucial to understanding the hemostatic and thrombotic process.

GPVI is a platelet receptor for collagen and is constitutively bound to the Fc receptor gamma chain, which contains an ITAM (28). Collagen engagement of GPVI results in Src family kinase (SFK) phosphorylation of the ITAM, to which Syk binds *via* its two SH2 domains as it binds to other ITAM-containing receptors (29, 30, 31, 32, 33). Syk is then phosphorylated by SFKs and subsequently by autophosphorylation. Activation of Syk in this manner allows phosphorylation of linker for T-cell activation (LAT) and SH2-domain–containing leukocyte protein of 76 kDa (SLP-76) and subsequent activation of PI3K. Bruton’s tyrosine kinase is also activated and recruited to the signaling complex that includes LAT and SLP-76 and phosphorylates phospholipase C γ2 (PLCγ2) (34, 35).

We previously showed that several tyrosine residues on Syk are phosphorylated in response to the GPVI agonist convulxin (36, 37). Studies in several cell types indicated that Y342 and Y346, located in the interdomain B (linker) region of Syk, are involved in the regulation of Syk activity and functions (36, 38, 39, 40, 41, 42). The molecular basis of their functions is not fully understood, but it appears likely that phosphorylation of these residues is involved in the transition of Syk from its autoinhibited conformation to its active conformation (43). Several studies indicate that phosphorylation of both Y342 and Y346 is critical for Syk activation (40, 41), but some data argue that Syk Y342 is the most important of the regulatory tyrosine residues in the interdomain B (39, 40, 41, 42), and the results of these studies vary for different cell types and responses. Some data indicate that the effects of pY342 and pY346 are specific (40). Using Syk Y342F knock-in mice, we recently demonstrated that phosphorylation of Syk Y342 plays an important positive regulatory role in the signal transduction from an ITAM receptor (44). We also showed that bleeding is unaffected in Syk Y342F mice while thrombus formation is significantly prolonged (44).

In this study, we focused on Syk Y346. We reported that Syk Y346 phosphorylation occurs upon GPVI stimulation independent of Syk activation. We then generated Syk Y346F knock-in mice to evaluate the role of this phosphorylation in downstream signaling and platelet functions. We report here that Syk Y346 phosphorylation negatively regulates Syk activity and downstream signaling events and platelet responses *in vitro*. Although the Syk activity is enhanced in Syk Y346F knock-in mouse platelets, hemostasis appears normal but the time to occlusion in the FeCl_3_ injury model is shortened.

## Results

### Phosphorylation of Syk tyrosine residues upon stimulation of ITAM receptors in platelets

**Figure 1:**
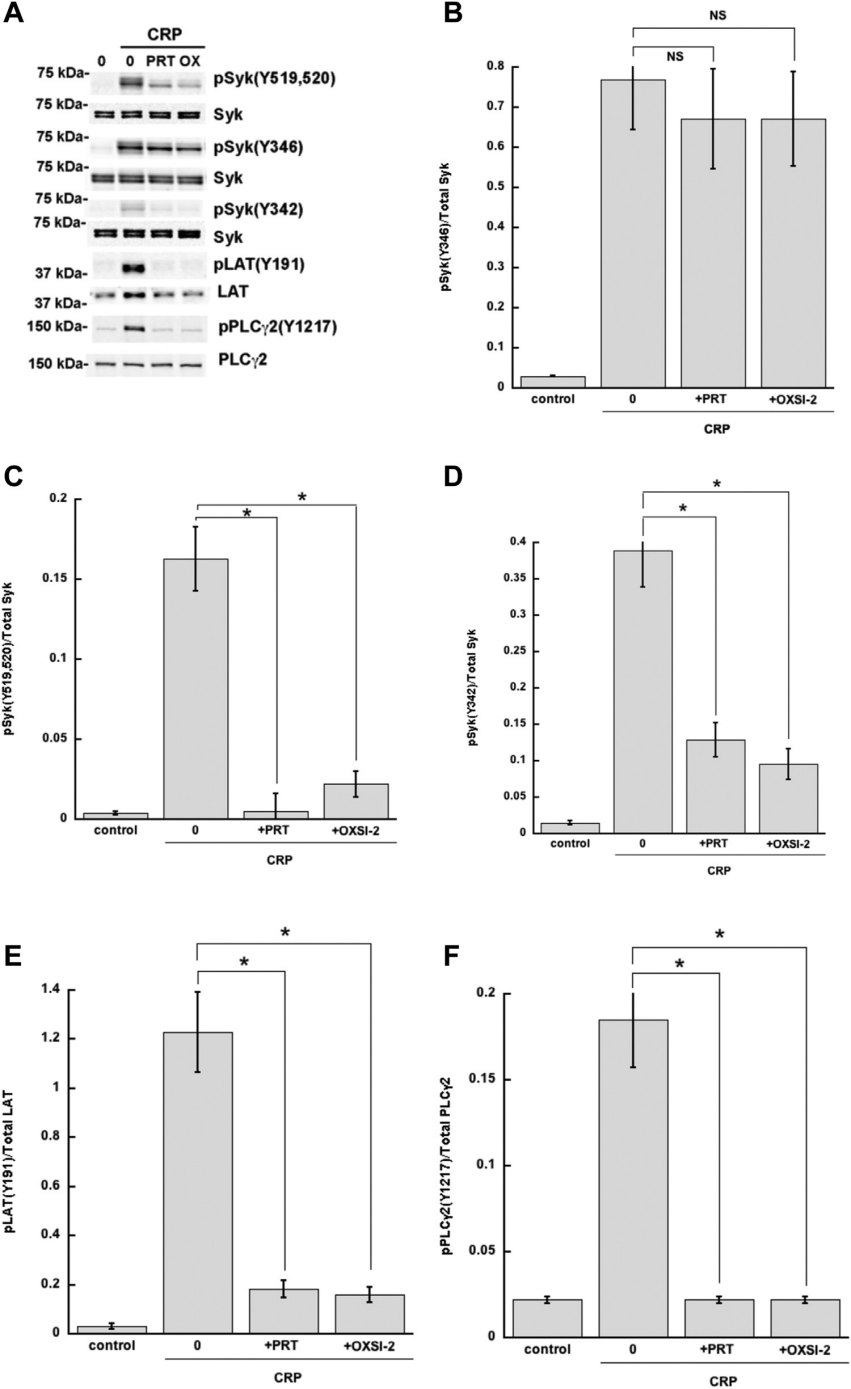
**Effect of Syk inhibitors on CRP-induced Syk tyrosine phosphorylation.** Platelets were isolated from WT mice and preincubated with either PRT062607 (1 μM) or OXSI-2 (1 μM) or vehicle for 5 min at 37 °C prior to activation with CRP (1 μg/ml). *A*, Western blot images showing phosphorylation of the indicated Syk tyrosine residue, PLCγ2 Y1217, LAT Y191, and the complimentary total protein. *B*–*F*, quantification of the indicated phosphorylated residue expressed as a ratio of the total protein. ∗*p* < 0.05, n = 4. CRP, collagen-related peptide; LAT, linker for T-cell activation; PLCγ2, phospholipase C γ2; Syk, spleen tyrosine kinase.

**Figure 2:**
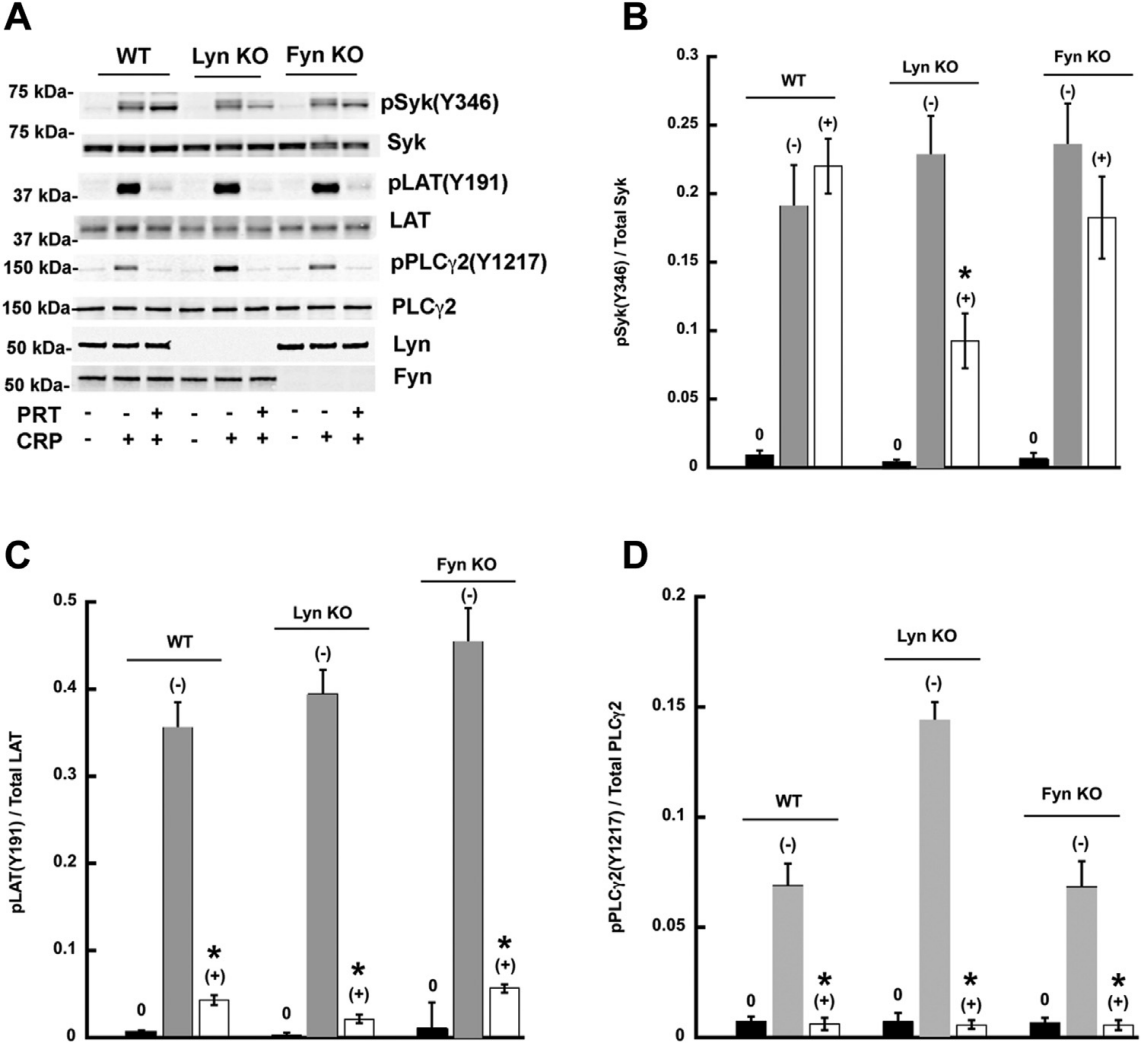
**Lyn is the major SFK responsible for the CRP-induced phosphorylation of Syk Y346.** Platelets were isolated from WT, Lyn KO, or Fyn KO mice and preincubated with vehicle or PRT062607 (1 μM) for 5 min prior to activation with CRP (1 μg/ml). *A*, Western blot images showing phosphorylation of Syk Y346, PLCγ2 Y1217, LAT Y191, and the complimentary total protein. Western blots of Fyn and Lyn were done to confirm that the SFK was absent. *B*–*D*, quantitation of the indicated phosphorylated residue expressed as a ratio of the total protein. Unstimulated (*black bars*), CRP preincubated with vehicle (*gray bars*), CRP preincubated with PRT (*white bars*). ∗*p* < 0.05, n = 3. CRP, collagen-related peptide; LAT, linker for T-cell activation; PLCγ2, phospholipase C γ2; SFK, Src family kinase; Syk, spleen tyrosine kinase.

Syk contains several tyrosine residues that can be phosphorylated, and of these, Y342, Y346, and Y519/520 have been suggested to play a role in the regulation of Syk activity (38, 39, 40, 41, 45, 46, 47, 48). To determine whether in our system these sites are phosphorylated by active Syk itself (autophosphorylation) or independently of that (by other kinases), we used the known Syk inhibitors PRT062607 (49) and OXSI-2 (50) to block the Syk activity thus eliminating autophosphorylation events. When mouse platelets were stimulated with the GPVI receptor agonist collagen-related peptide (CRP) in the presence of these Syk inhibitors, we observed that Y346 phosphorylation occurred even when Syk activity was blocked (Fig. 1, *A* and *B*). The phosphorylation of Y342 (Fig. 1*D*) and Y519/520 (Fig. 1*C*) was nearly abolished in the presence of Syk inhibitors in mouse platelets as was LAT (Fig. 1*E*) and PLCγ2 (Fig. 1*F*) phosphorylation. These data argue that Syk Y346 is directly phosphorylated by SFKs associated with the ITAM receptors. To ascertain the SFK responsible for the phosphorylation of Syk Y346, we activated Lyn KO platelets and Fyn KO platelets with CRP and measured Syk Y346 phosphorylation in the presence of PRT062607 (Fig. 2). Syk Y346 phosphorylation was significantly impaired in the CRP-activated Lyn KO platelets in the presence of PRT062607, indicating that Lyn plays a major role in phosphorylating this site (Fig. 2, *A* and *B*). Phosphorylation of LAT Y191 (Fig. 2*C*) and PLCγ2 Y1217 (Fig. 2*D*) was abolished demonstrating the efficiency of PRT062607 in inhibiting Syk activity. It is interesting to note that Syk Y346 phosphorylation is not abolished in the CRP-induced Lyn KO platelets without PRT062607. Syk tyrosine residues, including 346, are also autophosphorylated upon activation of Syk. In the absence of Lyn, the effect on the phosphorylation of Syk Y346 is masked by the autophosphorylation events triggered by the activation of Syk by other SFKs. In order to appreciate the difference between direct phosphorylation and autophosphorylation of Y346, the autophosphorylation events should be blocked and, hence, Syk was inhibited with PRT062607. While other SFKs might be able to phosphorylate Y346 in the absence of Lyn, Figure 2*B* shows that Lyn is the predominant kinase that directly phosphorylates Y346. Hence, in the Figure 1, Lyn can directly phosphorylate Y346, and hence we do not see much difference in the signal. In the Figure 2, autophosphorylation has to be inhibited to see the effect of Lyn on Y346. In both WT and Fyn null mice, the effect of PRT seems to be similar on Y346 phosphorylation (Fig. 2), similar to the Figure 1.

### Production and characterization of Syk Y346F knock-in mice

**Figure 3:**
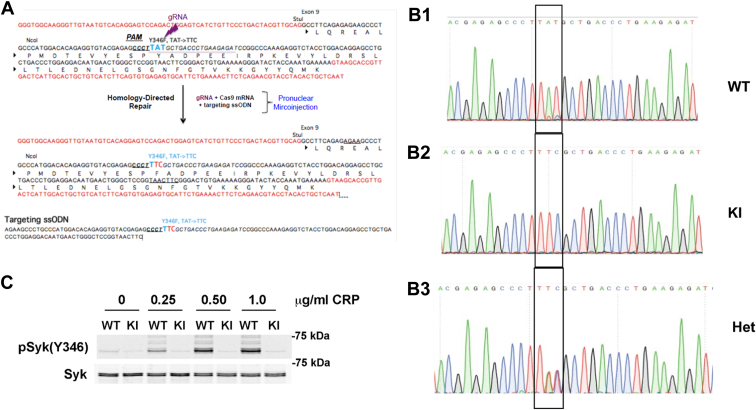
**Generation of Syk Y346F knock-in mice.***A*, the process of creating the mutant mouse *via* CRISPR is schematically presented. The targeted DNA codon is changed from TAT to TTC, changing the amino acid from tyrosine to phenylalanine. *B*, sequence analysis of PCR products of WT, Y346F homozygous, and WT/Y346F heterozygous (HET) mouse DNA. The oligonucleotides used for PCR are forward primer 5′- CTCCGCTGCATGCAACTGTC and reverse primer 5′-GCAGTGCAATGAGTCAACGGTGC. *C*, representative Western blot showing that Syk Y346 phosphorylation is present in CRP-stimulated WT platelets but absent in Syk Y346F knock-in platelets. CRP, collagen-related peptide; Syk, spleen tyrosine kinase; UN, unstimulated sample.

Studies in several cell types indicated that Syk Y342 and Y346, located in the interdomain B (linker) region, are involved in the regulation of Syk activity and functions (36, 38, 39, 40, 41, 42). We have recently demonstrated that Y342 phosphorylation positively regulates Syk activity in platelets (44). To determine the function of Y346 in ITAM receptor–mediated signaling and regulation of Syk, we produced a Syk Y346F knock-in mouse using the CRSPR/Cas9 technique (Fig. 3*A*) through a commercial source (Cyagen). The Syk Y346F mutation was confirmed by performing PCR with a pair of specific primers followed by sequencing of the PCR products (Fig. 3*B*). WT mice demonstrated the TAT sequence corresponding to a tyrosine codon (Fig. 3*B*1), the homozygous mutant mice exhibited the TTC sequence corresponding to a phenylalanine codon (Fig. 3*B*3), and the heterozygous mice expectedly showed the T(T/A)(T/C) sequence (Fig. 3*B*2). To confirm the Y346F mutation at the protein level, we isolated platelets from Syk Y346F and WT littermate control mice and stimulated them with CRP. We performed Western blot analysis to visualize phosphorylation of Y346 and observed a robust band in WT mouse platelets treated with CRP at any concentration but no band at the expected molecular weight in samples obtained from Syk Y346F mouse platelets (Fig. 3*C*). Taken together, these data demonstrate that we successfully produced Syk Y346F knock-in mice.

Both heterozygous Syk Y346F knock-in mice and homozygous Syk Y346F knock-in mice bred normally and produced pups at expected Mendelian ratios. Blood cell counts were not altered in Syk Y346F knock-in mice (Table 1).

**Table 1:** Blood cell counts from Syk Y346F and WT littermate control mice

Sample	WBC(K/ml)	NE (K/ml)	LY (K/ml)	RBC (M/ml)	PLT (K/ml)	MPV (fL)
WT	9.54 ± 1.4	0.95 ± 0.18	9.06 ± 0.43	10.11 ± 0.27	732.6 ± 57.7	4.47 ± 0.12
Syk (Y346F)	8.40 ± 1.9	0.81 ± 0.07	7.72 ± 0.77	9.85 ± 0.14	716.2 ± 38.1	4.27 ± 0.06

Abbreviations: LY, lymphocyte; MpV, mean platelet volume; NE, neutrophil; PLT, platelet; RBC, red blood cell; WBC, white blood cell.

### Syk Y346F platelets show enhanced GPVI-mediated aggregation and secretion

**Figure 4:**
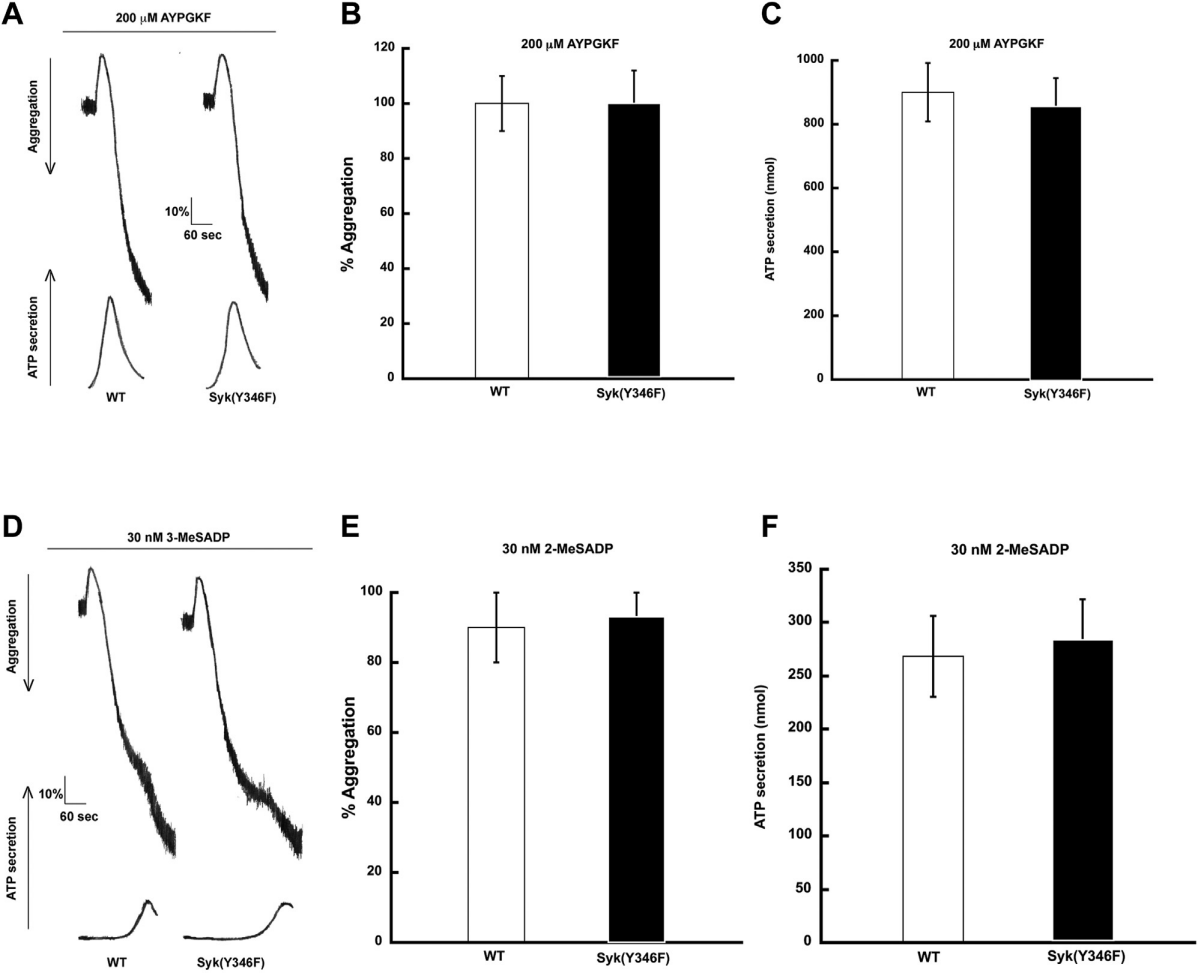
**GPCR-mediated platelet reactivity is intact in Syk Y346F mice.***A*, representative aggregation and secretion tracings of platelets from WT and Syk Y346F mice activated with the PAR4 agonist AYPGKF. *B*, quantification of aggregation and (*C*) ATP secretion of platelets from Syk Y346F and WT littermate control mice stimulated with 200 μM AYPGKF. *D*, representative aggregation and secretion tracings of platelets from WT and Syk Y346F mice stimulated with the P2Y12 agonist 2-MeSADP. *E*, quantitation of aggregation and (*F*) ATP secretion of platelets from Syk Y346F and WT mice stimulated with 30 nM 2-MeSADP. n = 4. GPCR, G protein-coupled receptor; Syk, spleen tyrosine kinase.

**Figure 5:**
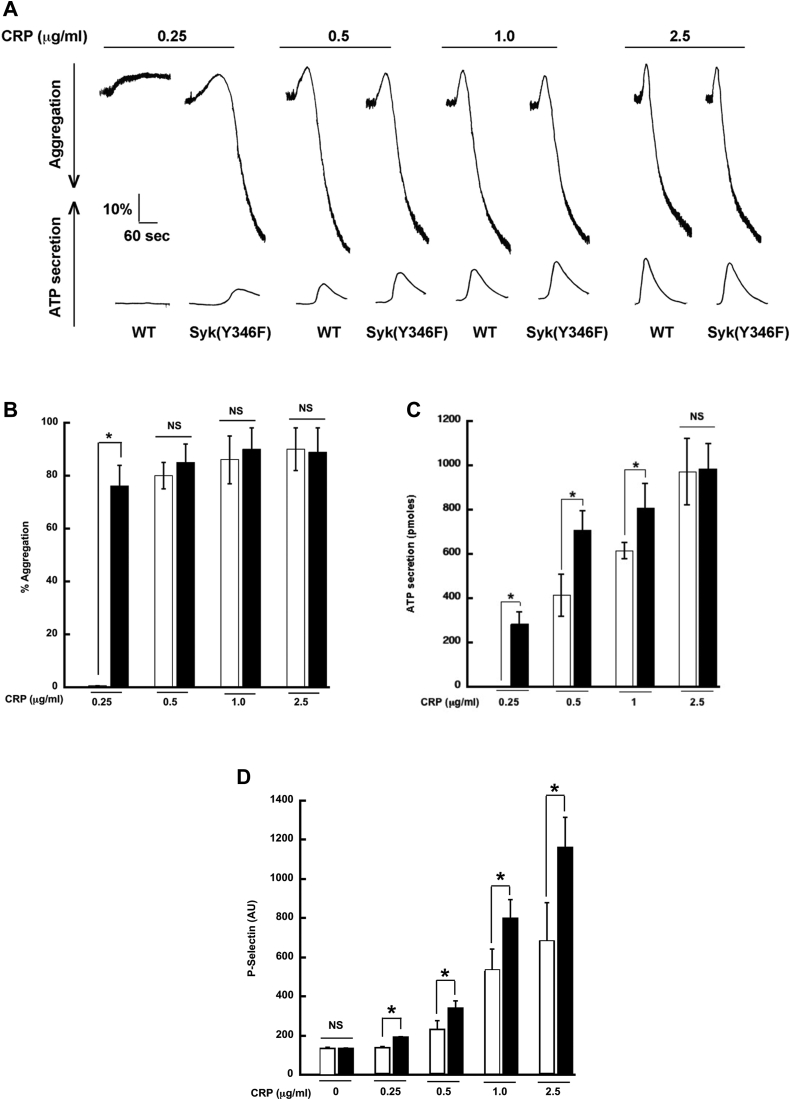
**GPVI-mediated aggregation and secretion are potentiated in Syk Y346F platelets.***A*, representative aggregation and secretion tracings of Syk Y346F (*black bars*) and WT littermate control (*white bars*) platelets stimulated with the indicated concentrations of CRP. *B*, quantification of aggregation, (*C*) ATP secretion, and (*D*) P-selectin expression from multiple independent experiments. ∗*p* < 0.05, n = 3. CRP, collagen-related peptide; GPVI, glycoprotein VI; Syk, spleen tyrosine kinase.

Platelets from Syk Y346F mice respond normally to the PAR-4 agonist AYPGKF (Fig. 4, *A*–*C*) or the purinergic agonist 2-MeSADP (Fig. 4, *D*–*F*); this result is consistent with the lack of critical involvement of Syk in signaling through these platelet receptors. Because Syk activity is vital to platelet activation by GPVI agonists, we evaluated the effect of Syk Y346F on GPVI-mediated aggregation and secretion by activating platelets isolated from Syk Y346F and WT littermate control mice with varying concentrations of CRP. Our results indicated that there was no aggregation (Fig. 5, *A* and *B*) or ATP secretion (Fig. 5, *A* and *C*) in WT platelets at very low concentrations of CRP, whereas Syk Y346F platelets aggregated and secreted under these conditions. Furthermore, Syk Y346F platelets showed a significant increase in ATP secretion at the moderate concentrations of CRP compared to platelets from WT littermates. This enhanced response was not observed when a high concentration of CRP was used (Fig. 5, *A*–*C*). We also evaluated alpha granule release by flow cytometry using P-selectin as a marker and demonstrated that alpha granule release was increased in Syk Y346F platelets compared to WT control platelets (Fig. 5*D*).

### GPVI-mediated signaling is enhanced in Syk Y346F platelets

**Figure 6:**
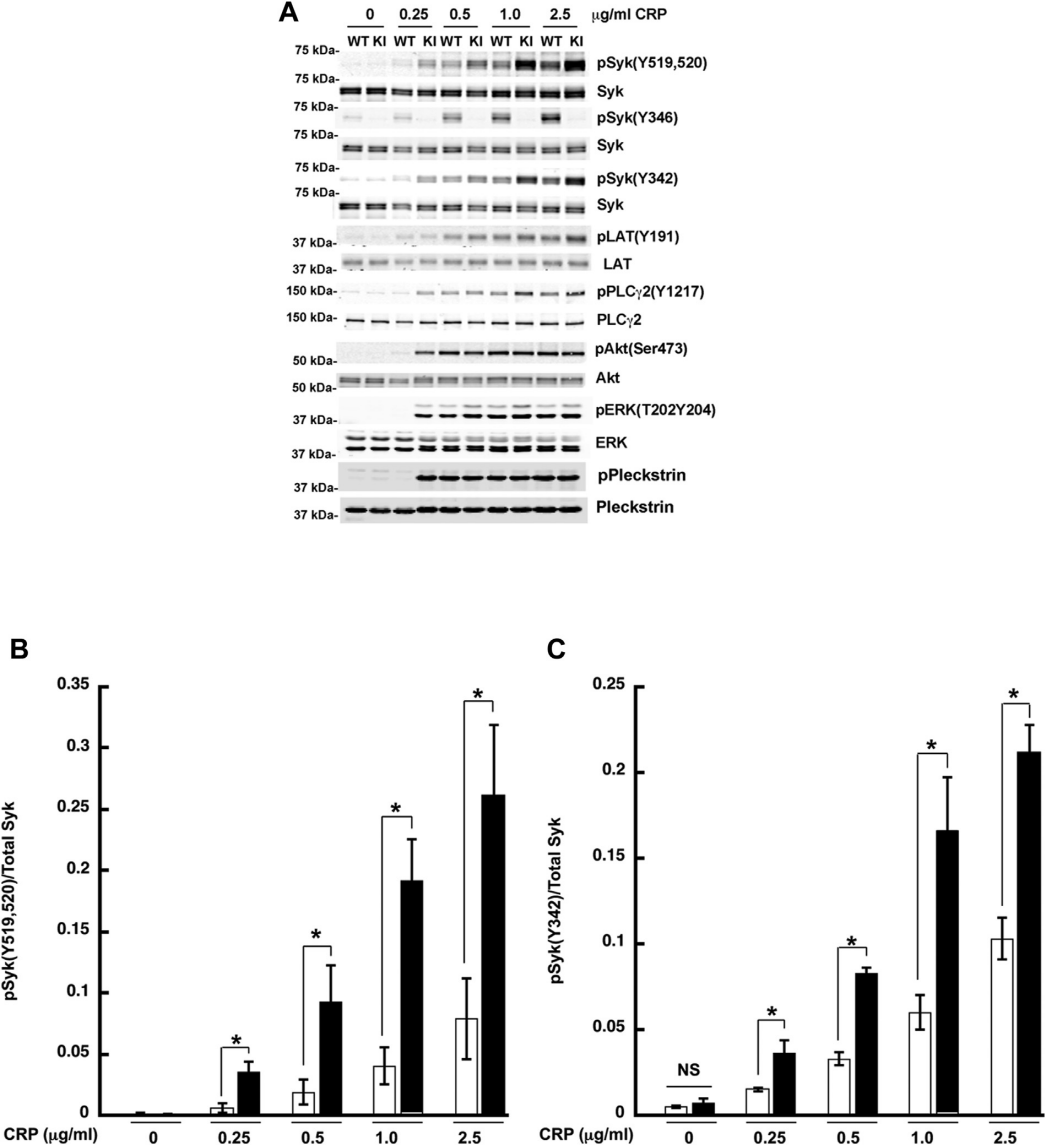

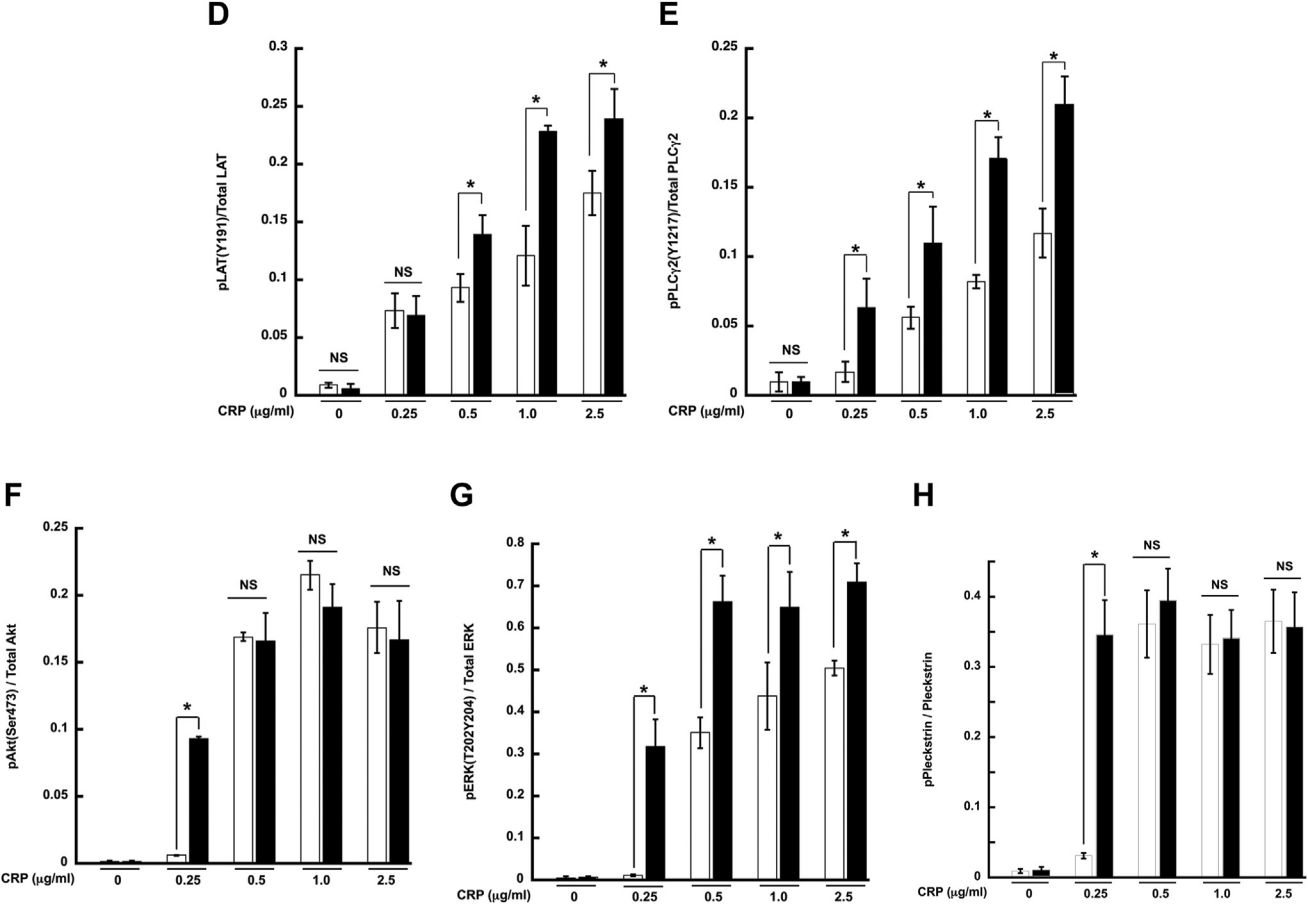
**CRP-mediated signaling is increased in Syk Y346F platelets.***A*, representative Western blots showing the indicated phosphorylated and total protein in Syk Y346F and WT littermate control platelets stimulated with the indicated concentrations of CRP for 3 min. *B*–*H*, quantification of the indicated phosphorylated residue expressed as a ratio of the total protein. ∗*p* < 0.05, n = 5. CRP, collagen-related peptide; Syk, spleen tyrosine kinase.

Syk is an essential component of the signaling pathway downstream from ITAM-bearing receptors (30, 31, 32, 33). Therefore, we analyzed phosphorylation of key proteins involved in the signaling cascade downstream of GPVI stimulation. After activation with low concentrations of CRP, under the conditions of no aggregation or secretion, there is no phosphorylation of Syk Y519/520, Syk Y342, LAT191, or PLCγ2 Y1217 in WT platelets, but phosphorylation of all these sites is dramatically enhanced in Syk Y346F platelets (Fig. 6, *A*–*E*). Contrary to the aggregation and secretion data, the phosphorylation of Syk Y519/520, Syk Y342, LAT Y191, or PLCγ2 Y1217 was enhanced at all concentrations of CRP tested. Extracellular signal-regulated kinase (ERK) phosphorylation was also enhanced in Syk Y346F platelets at all CRP concentrations tested (Fig. 6*G*), while Akt (Protein kinase B) pSer473 and pleckstrin phosphorylation mimicked the aggregation pattern (Fig. 6, *F* and *H*). These data collectively indicate that signaling downstream of GPVI is enhanced in Syk Y346F platelets.

We have evaluated the effect of pan-Src kinase inhibitor PP2, and the data are shown in Fig. S1. As can be seen in Fig. S1, all phosphorylations are wiped out by pan-Src inhibitor PP2 in WT and Y346F mutant mice. We also evaluated a possibility that zeta-chain–associated protein kinase 70 (ZAP-70), an SFK similar to Syk, has a redundant role in GPVI-mediated signaling of platelets. As shown in the Fig. S2, platelets and B cells do not express ZAP-70. This finding rules out the possibility of ZAP-70, mediating the effects in platelets. We have further evaluated the surface levels of GPVI on platelets from WT and Syk Y346F mutant mice using flow cytometry. The results, shown in Fig. S3, indicate that there is no difference in the surface level of GPVI between the WT and Y346F mouse platelets. Hence, the negative regulatory effect seen in Y346F mice is not due to an enhanced surface expression of GPVI receptors.

### HemITAM-mediated aggregation and secretion is enhanced in Syk Y346F platelets

**Figure 7:**
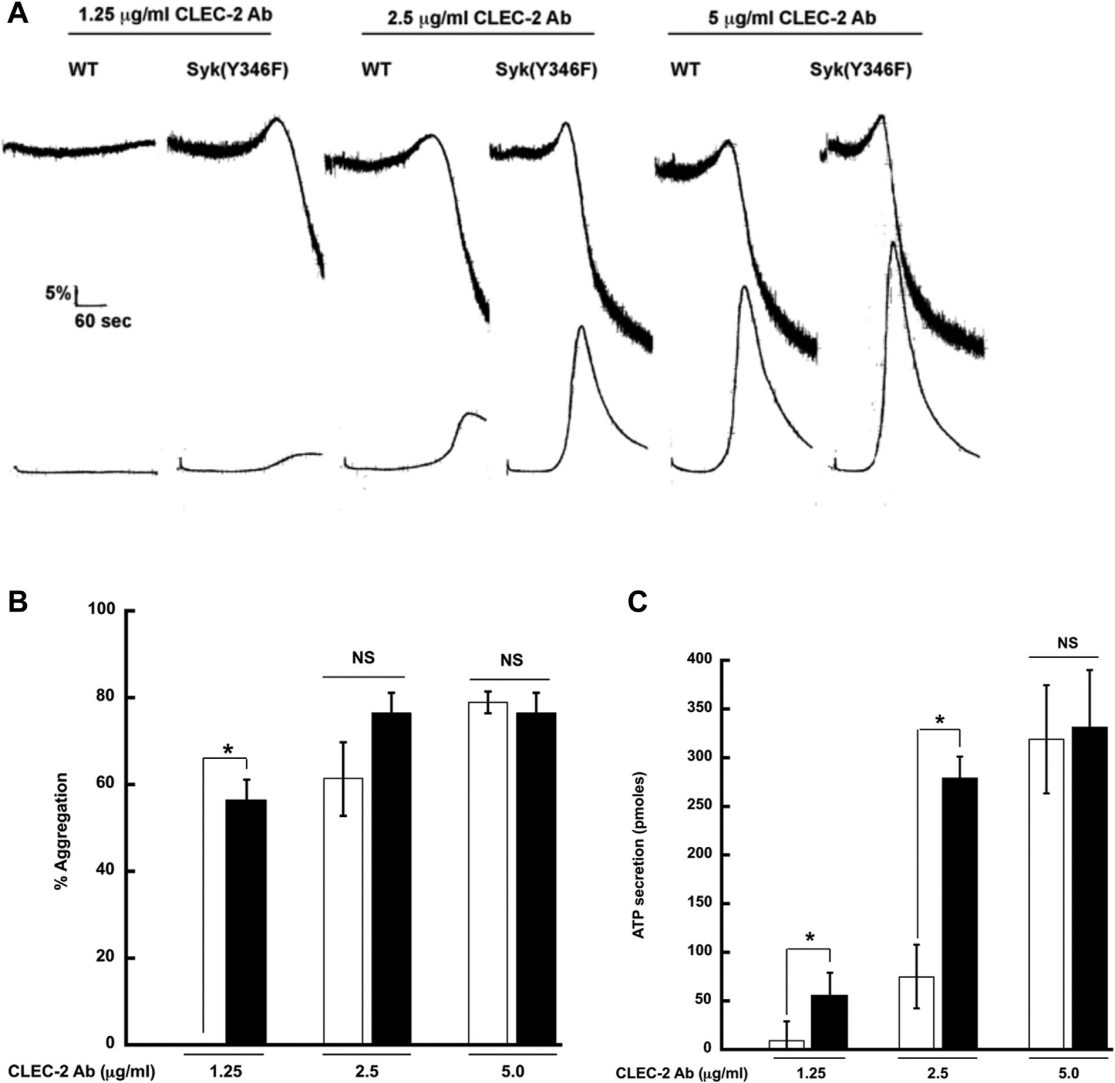
**CLEC-2–mediated aggregation and secretion are potentiated in Syk Y346F platelets.***A*, representative aggregation and secretion tracings of Syk Y346F (*black bars*) and WT littermate control (*white bars*) platelets stimulated with the indicated concentrations of CLEC-2 antibody. *B*, quantification of aggregation and (*C*) ATP secretion from multiple independent experiments. ∗*p* < 0.05, n = 5. CLEC, C-type lectin-like receptor; Syk, spleen tyrosine kinase.

Syk is also crucial for hemITAM-dependent signaling initiated through the CLEC-2 receptor (23, 51, 52, 53). We investigated whether there was a similar effect of the Syk Y346F mutation on CLEC-2–activated responses. We thus stimulated platelets isolated from Syk Y346F and WT littermate control mice with an antibody that crosslinks the CLEC-2 receptor and examined aggregation and ATP secretion. Similar to the effect we saw when the GPVI receptor was activated, potentiation of responses was seen in the Syk Y346F platelets compared to the WT platelets. When a high concentration of the CLEC-2 antibody was used, however, there was a robust aggregation in both (Fig. 7, *A*–*C*).

### HemITAM-mediated signaling is enhanced in Syk Y346F platelets

**Figure 8:**
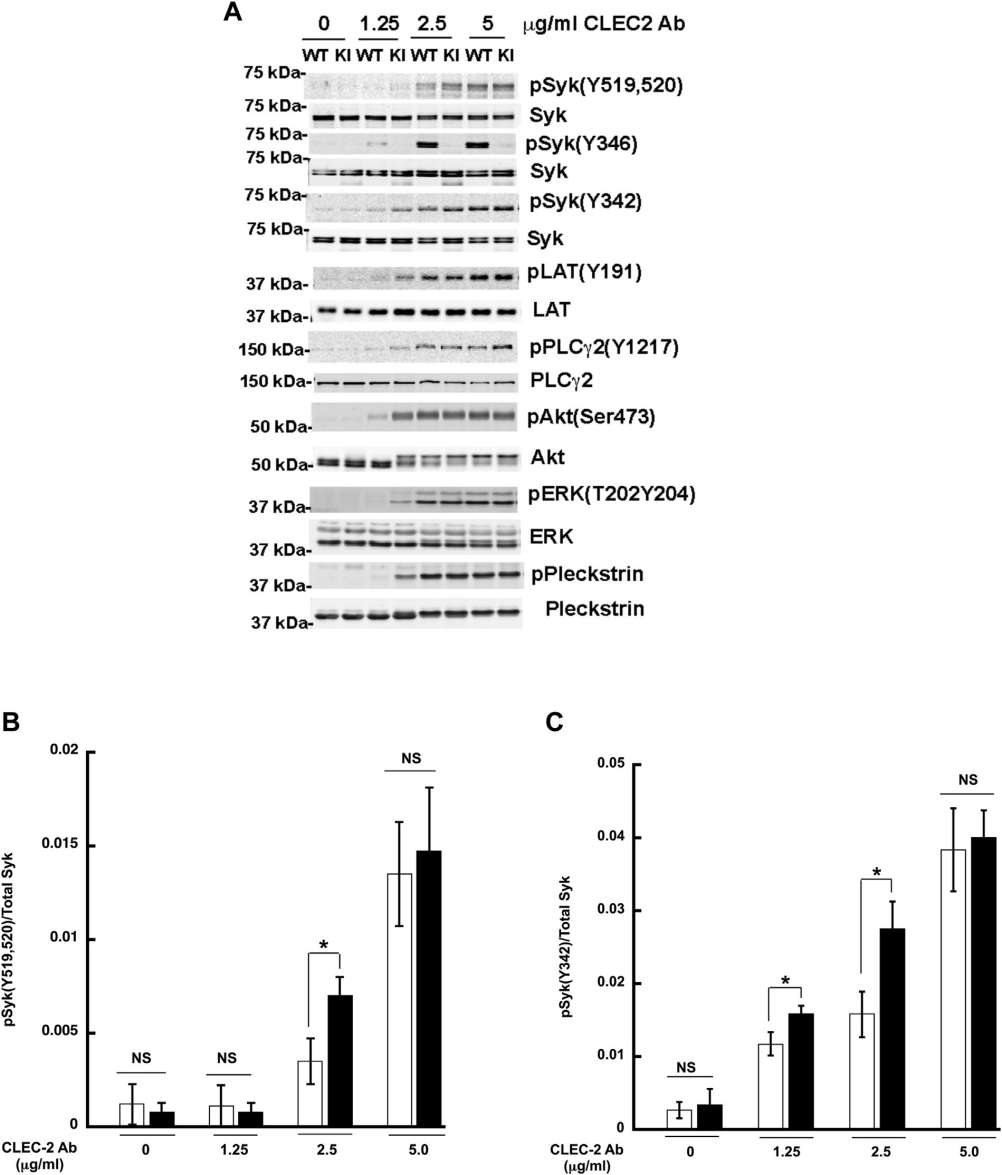

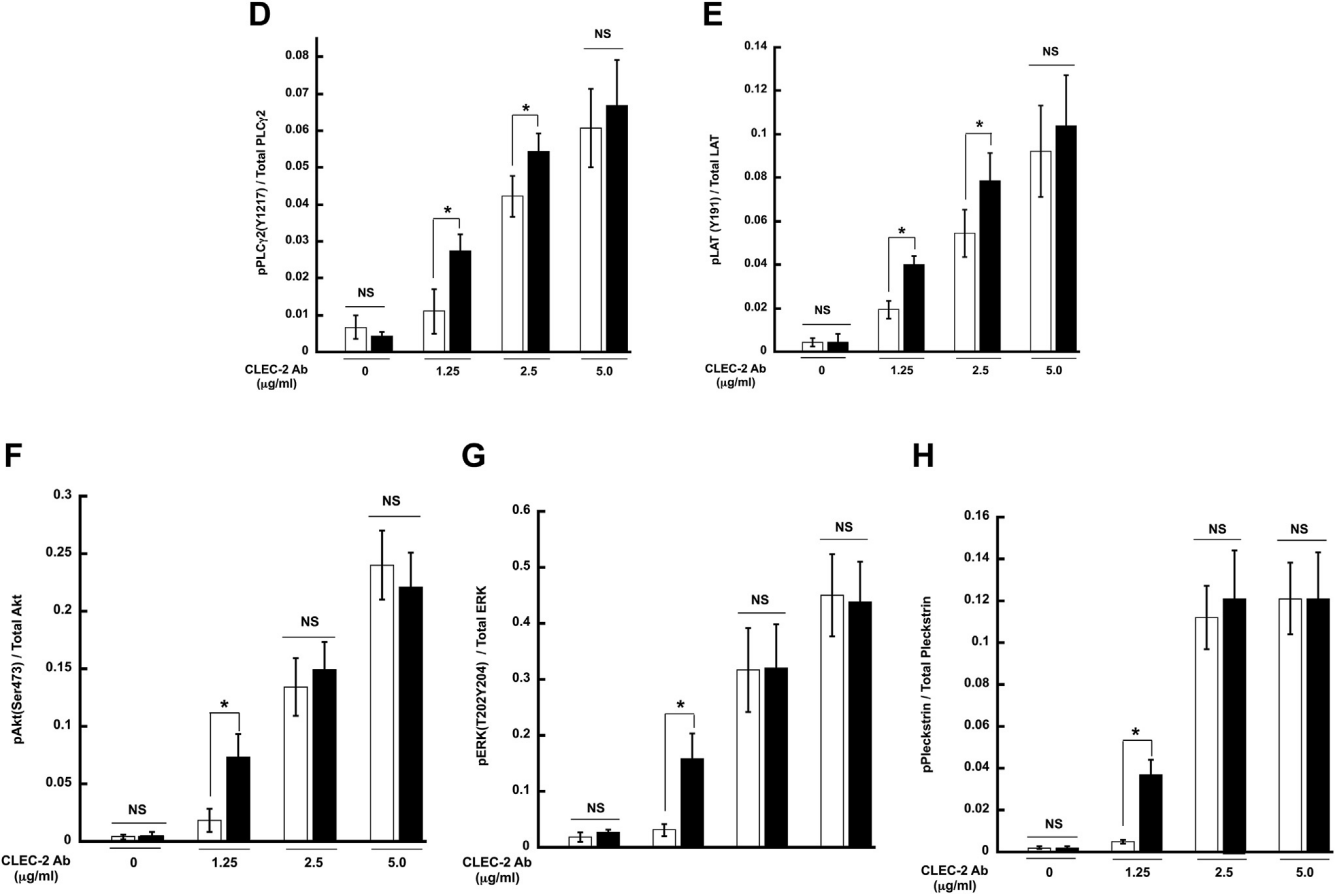
**CLEC-2–mediated signaling is increased in Syk Y346F platelets.***A*, representative Western blots depicting the indicated phosphorylated and total protein in Syk Y346F and WT littermate control platelets stimulated with various concentrations of CLEC-2 antibody as shown. *B*–*H*, quantification of the indicated phosphorylated residue expressed as a ratio of the total protein. ∗*p* < 0.05, n = 4. CLEC, C-type lectin-like receptor; Syk, spleen tyrosine kinase.

Since Syk is also a crucial component of the signaling pathway downstream from hemITAM-bearing receptors, we analyzed similar proteins as we did with GPVI stimulation. After activation of the CLEC-2 receptor, there was phosphorylation of Syk Y519,520, Syk Y342, LAT Y191, and PLCγ2 Y1217 in WT platelets, but the phosphorylation of these sites was statistically increased in the Syk Y346F platelets at lower concentrations of agonist (Fig. 8, *A*–*E*). We also evaluated the effect of Y346 phosphorylation on the Akt and ERK pathways. As seen in Figure 7, *A*, *E*, and F, Akt and ERK phosphorylation was increased in the Syk Y346F platelets compared to the WT control platelets at lower concentrations of the CLEC-2 agonist. Pleckstrin phosphorylation, indicative of calcium mobilization, was also increased in the CLEC-2–activated Syk Y346F platelets.

### Syk Y346 is not important for hemostasis but affects *in vivo* thrombus formation

**Figure 9:**
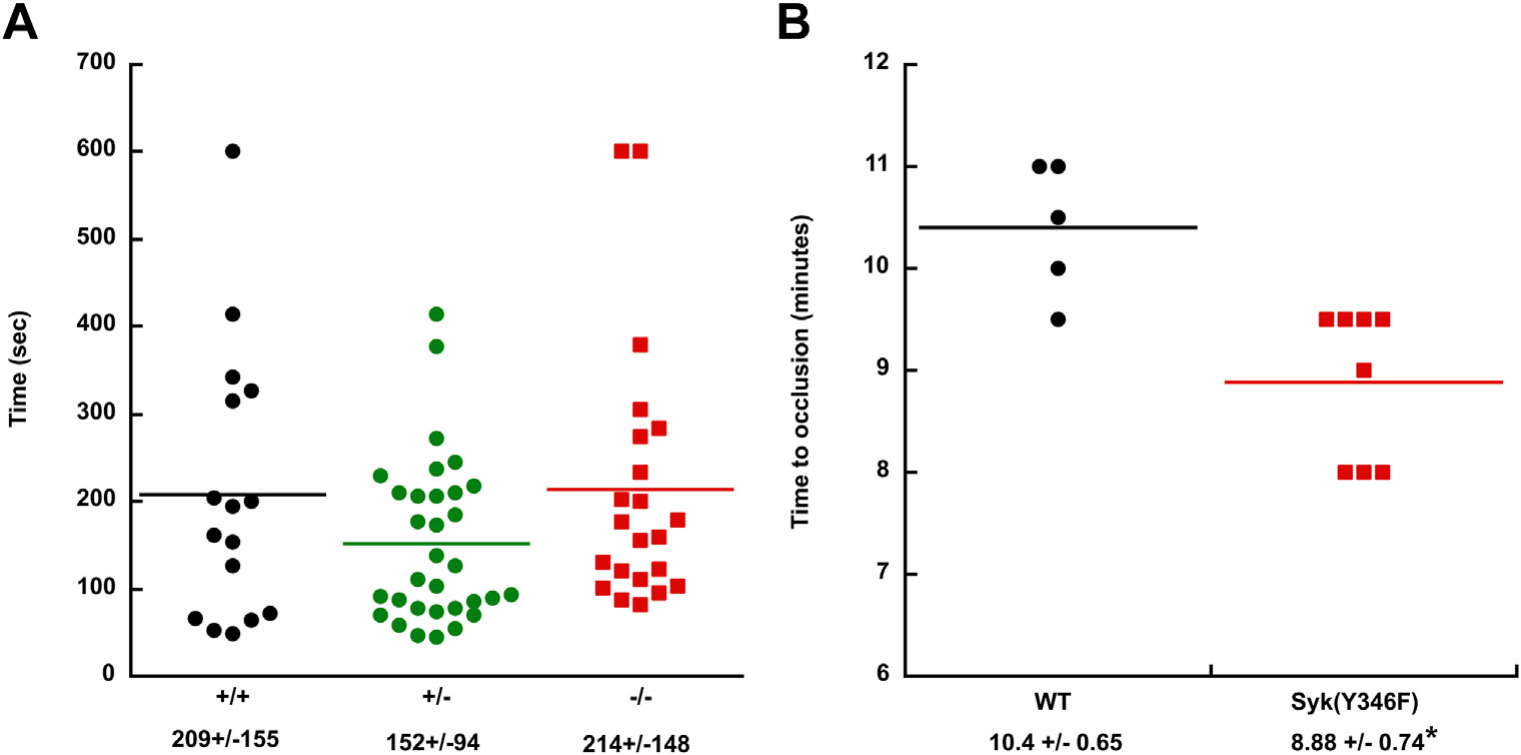
***In vivo* thrombus formation is enhanced in Syk Y346F knock-in mice.***A*, scatter plot showing the time it took for bleeding to stop during tail-bleeding experiments conducted on homozygous Syk^Y346F/Y346F^ knock-in, heterozygous Syk^Y346/WT^, and WT (Syk^WT/WT^) littermate control mice 4 to 6 weeks of age in a blind fashion. *B*, scatter plot of the time to occlusion in WT and Syk Y346F knock-in mice following 5% FeCl_3_ injury on the carotid artery. Syk, Spleen tyrosine kinase.

To determine the impact of Syk Y346F on hemostasis, we performed tail-bleeding assay on litter-matched WT, heterozygous, and homozygous Syk Y346F mice. We found no significant differences in the bleeding times (Fig. 9*A*). These data suggest that Syk Y346 phosphorylation exerts no effect on hemostasis. To determine whether thrombus formation is impacted by the impaired phosphorylation of Y346 on Syk, we injured the carotid artery in Syk Y346F and WT littermate control mice with 5% FeCl_3_ for 90 s and monitored its occlusion. Syk Y346F mice formed stable occlusion in statistically shorter time than the WT mice (Fig. 9*B*). These data suggest that the positive effect of the Syk Y346F mutation we observed for GPVI and CLEC-2 mediated signaling and platelet responses *in vitro* moderately but significantly potentiates thrombus formation *in vivo*.

## Discussion

It has been known that Syk is phosphorylated on multiple tyrosine residues and many of these residues exert regulatory effects on Syk activity and Syk-mediated signaling (15, 16, 18, 19, 33). However, specific contributions of the individual tyrosine phosphorylation (pY) sites to this process require elucidation. Among the pY-sites of Syk, pY346 is of great interest for two reasons; it had been argued previously that this site (i) exerts a positive effect on Syk activation and signaling (40, 41) and (ii) is phosphorylated by SFKs independently of Syk activity (54). Both features make this site potentially important for the regulation of Syk-mediated signaling.

Phosphorylation of Syk Y346 by SFKs, of which Lyn appeared to be the most important, has been shown in mouse bone marrow–derived mast cells and the RBL-2H3 rat basophil/mast cell line (54). We have examined the specificity of Y346 phosphorylation in platelets using selective inhibitors of Syk and SFK KOs and demonstrated that while pY342 and pY519/520 are mostly products of autophosphorylation, formation of pY346 is mostly independent of Syk autophosphorylation and at least in part depends on Lyn (Figs. 1 and 2). These results are consistent with the idea that Y346 is phosphorylated mostly by SFKs, thus representing a major target of SFK-mediated regulation of Syk activity following engagement of ITAM-bearing receptors. The key role of SFKs in the initial events of ITAM-bearing receptor signaling had been shown in multiple systems (8, 10, 55, 56, 57), but the targets of SFK-dependent regulation on Syk remained largely unknown. Our results together with the data on mast cells (54) firmly establish Syk Y346 as a phosphorylation site for SFKs.

The notion of a positive effect of Syk pY346 on Syk activity is based on the data of several studies. Thus, Syk Y346F mutation reduced tyrosine phosphorylation of such key signaling proteins as PLC-γ and LAT and, especially, phosphorylation of Erk and Akt protein kinases in Syk-null mouse mast cells reconstituted with WT or mutant Syk (40), albeit the effect of this mutation on antigen-induced degranulation of the Syk-reconstituted mouse primary cells, and the RBL-2H3 rat basophilic/mast cell line was moderate (39, 40). Syk Y346F also reduced activation of the transcription factor NF-AT in the Syk-reconstituted DT40 chicken B-cell line stimulated through the B-cell receptor (41). However, the role of Y346 had been studied mostly in mutant Syk lacking several pY-sites; in many studies, only the mutation involving both Y342 and Y346 had been examined (38, 40, 41, 45). Notably, when the single Y346F mutation could be compared to the double YY342/346FF mutation, the latter exerted a much stronger effect on signaling and cell activation (40, 41). Overall, the specific regulatory role of Syk Y346 remained uncertain, especially in platelets, where mutational analysis of Syk Y346 had never been performed. The results reported in this study indicate that platelets from Syk Y346F mice are hyperreactive to stimulation through the GPVI receptor for collagen. Although aggregation, secretion, and degranulation of WT and Syk Y346F platelets *in vitro* demonstrate somewhat differential dependence on a concentration of CRP, a GPVI receptor agonist, all responses are clearly upregulated at a suboptimal stimulation level (Fig. 5). These results are consistent with the data on GPVI-mediated signaling; the lack of Syk pY346 increases Syk autophosphorylation and phosphorylation of key signaling proteins (Fig. 6). Similar results were obtained with CLEC-2 receptor clustering (Figs. 7 and 8). Together, our results argue that Syk pY346 negatively regulates Syk activity and Syk-dependent signaling and cellular responses in platelets upon the GPVI receptor or CLEC-2 engagement.

A difference between our results and the notion of Syk pY346 acting as a positive regulatory site (40, 41) may be explained by the disparity between the experimental systems utilized; previous studies have been performed either in Syk-negative B-cell or basophil/mast cell lines reconstituted with WT or mutant Syk or with isolated Syk *in vitro*, whereas ours are based on primary cells from a knock-in mouse naturally expressing a mutant form of Syk. Another possible explanation of the observed differences is provided by the cell type–specific factors of Syk-mediated signaling and regulation, such as activating receptors, Syk-binding proteins, Syk substrates, and regulatory proteins. For example, the downregulatory effect of T-cell ubiquitin ligand-2 (TULA-2) protein tyrosine phosphatase on Syk activation and Syk-mediated signaling in platelets appears to exceed that in other cells where TULA-2 downregulates Syk signaling, such as basophils/mast cells (58) and osteoclasts (59), in agreement with a very high level of TULA-2 in platelets exceeding that in other hematopoietic cells (60). Considering that pY346 appears to be the most favored target of TULA-2 in Syk (36), Y346F mutation may upregulate Syk in platelets by abolishing a major mechanism of its negative regulation.

Furthermore, it should be noted that Syk activity and Syk regulation may be affected by a mutation of its pY-site in a complex, multifaceted way. Thus, it has been shown that the kinase activity of purified unphosphorylated Syk *in vitro* is significantly upregulated by Y-to-F mutations of various sites located throughout the Syk molecule, including Y352 (a human Syk residue corresponding to mouse Y346) and even Y525/526 (a human homolog of mouse Syk Y519/520) (61), whose key role in Syk activity has been demonstrated in very diverse biological systems (46, 47, 48). Despite the apparent positive effect of the Y352F mutation on basal activity of unphosphorylated Syk in this system, which is consistent with our results reported here, this mutation prevents *in vitro* activation of Syk by pITAM while not affecting the activation of Syk by Lyn. In contrast, no effect is exerted by Y352F on either basal, or pITAM-, or Lyn-enhanced activity of Syk, if Syk is preincubated with ATP to become phosphorylated prior to *in vitro* kinase assay (61).

Overall, the apparent differences between the positive effect of Syk Y346F mutation on Syk activity and signaling in mouse platelets that we report here and the effects of this mutation in some other systems may be due to the different cellular context, especially considering the likely mechanistical complexity of the pY346 contribution to the regulation of Syk activity. It is possible that one of the consequences of such complexity is the lack of a dramatic effect of Syk Y346F mutation *in vivo* (Fig. 8) despite its clear positive effect on GPVI signaling and GPVI-dependent platelet responses *in vitro*.

## Experimental procedures

### Antibodies and reagents

All reagents were purchased from Thermo Fisher Scientific unless otherwise stated. Chrono-lume, used for the detection of secreted ATP, was purchased from Chrono-log corporation. Anti-pSyk Y525/526 (mouse Y519/520) and anti-pPLCγ2 Y1217 were purchased from Cell Signaling Technology. Anti-pSyk Y352 (Y346 in mice) and anti-pSyk Y348 (Y342 in mice) were purchased from Abcam. Anti-Syk and anti-PLCγ2 were purchased from Santa Cruz Biotechnology. Ibrutinib was purchased from Selleckchem. Odyssey blocking buffer and secondary antibodies IRDye 800CW goat anti-rabbit and IRDye 680LT goat anti-mouse were purchased from Li-Cor. CRP-XL was purchased from Dr Richard Farndale at the University of Cambridge. AYPGKF was purchased from GenScript.

### Animal housing and production

Mice were housed in a pathogen-free facility, and all animal procedures were approved by the Temple University Institutional Animal Care and Use Committee (protocol #4864). Syk Y346F mice were produced by Cyagen on a fee for service basis.

### Preparation of mouse platelets

Mouse blood was collected and platelets were isolated as previously described (62). The resulting platelets were counted using a Hemavet 950FS blood cell analyzer (Drew Scientific). Platelet counts were adjusted to a final concentration of 1.5 × 10^8^ cells/ml in N-2-hydroxyethylpiperazine-N′-2-ethanesulfonic acid-buffered (pH 7.4) Tyrode’s solution containing 0.2 U/ml apyrase.

### Platelet aggregation and ATP secretion

All platelet aggregation and secretion experiments were carried out using a lumi-aggregometer (Chrono-log) at 37 °C under stirring conditions. Platelet aggregation was measured using light transmission, and ATP secretion was measured using Chrono-lume (a luciferin/luciferase assay).

### Western blotting

Western blotting procedures were performed as described previously (62). Briefly, platelets were stimulated for the indicated time points in a lumi-aggregometer with a GPVI agonist. The reaction was stopped by precipitating the platelet proteins using 0.6 N HClO_4_ and washed with water prior to the addition of sample loading buffer. Platelet protein samples were then boiled for 5 min prior to resolution by SDS-PAGE and transfer to nitrocellulose membranes. The membranes were then blocked using Odyssey blocking buffer and incubated overnight with primary antibodies against the indicated protein. The membranes were then washed with Tris-buffered saline containing 0.1% Tween-20 prior to incubation with appropriate secondary antibodies for 1 h at room temperature (RT). The membranes were washed again and imaged using a Li-Cor Odyssey infrared imaging system.

### Flow cytometry

Surface exposure of P-selectin in murine platelets was determined as previously described (63). Briefly, aliquots (0.3 ml) of washed murine platelets were preincubated with the indicated concentrations of CRP for 5 min at 37 °C. An aliquot containing 10^6^ platelets was gently mixed with 10 μl FITC-anti-P-selectin (Emfret) and incubated for 15 min at RT. Platelets were fixed with 1% paraformaldehyde in PBS.

For surface expression of GPVI (clone JAQ1; Emfret) and αIIβ (CD41), aliquots of murine washed platelets were incubated with 10 μl of the corresponding antibody for 15 min at RT and treated as above. All determinations were performed on a FACSymphony flow cytometer (BD Scientific).

### Tail-bleeding assay

Mouse tail bleeding was conducted as previously described (64). Mice aged 4 to 6 weeks were anesthetized prior to amputation of the distal 3 mm of the tail. The tail was then immersed in 37 °C saline, and bleeding was monitored. If bleeding continued for greater than 600 s then the bleeding was halted manually by applying pressure.

### Carotid artery injury

FeCl_3_ was used to injure the carotid artery as previously described (64). Mice aged 10 to 12 weeks were anesthetized and the carotid artery was exposed. A baseline blood flow reading was obtained using a Transonic T402 flow meter. The carotid artery was injured using a 1 × 1 mm piece of filter paper saturated with 5% FeCl_3_ for 90 s. The filter paper was removed, and blood flow was recorded.

### Statistics

All statistical analysis was performed using Microsoft Excel, and data were analyzed using a Student’s *t* test where *p* < 0.05 was considered statistically significant. All the data are presented as means ± SD of at least three independent experiments.

## Data availability

All data concerning this report are available in the manuscript.

## Supporting information

This article contains supporting information.

## Conflict of interest

The authors declare that they have no conflicts of interest with the contents of this article.
