# Supplementary material for: Phosphorylation of spleen tyrosine kinase at Y346 negatively regulates ITAM-mediated signaling and function in platelets
Source: J Biol Chem. 2023 Jun 1;299(7):104865. doi: 10.1016/j.jbc.2023.104865 (PMC10320515; doi:10.1016/j.jbc.2023.104865)
Supplement: Supporting Figure S1 [file mmc1.pdf]

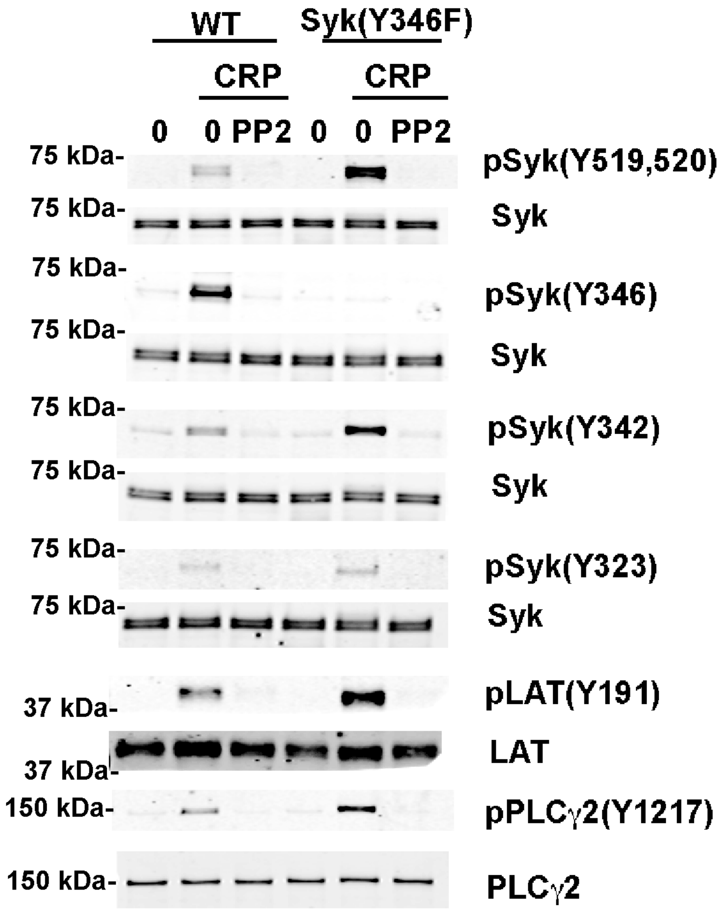

Figure S1

PP2 inhibits CRP-induced signaling.

Platelets were isolated from WT and Syk Y346F mice and pre-incubated with 10 μM PP2 or vehicle for 5 minutes at 37°C. Platelets were then activated with 1 μg/ml CRP for 60 seconds. Proteins were isolated , run on SDS-PAGE, transferred to nitrocellulose and probed for indicated phosphoproteins.
