# Supplementary material for: Phosphorylation of spleen tyrosine kinase at Y346 negatively regulates ITAM-mediated signaling and function in platelets
Source: J Biol Chem. 2023 Jun 1;299(7):104865. doi: 10.1016/j.jbc.2023.104865 (PMC10320515; doi:10.1016/j.jbc.2023.104865)
Supplement: Supporting Figure S2 [file mmc2.pdf]

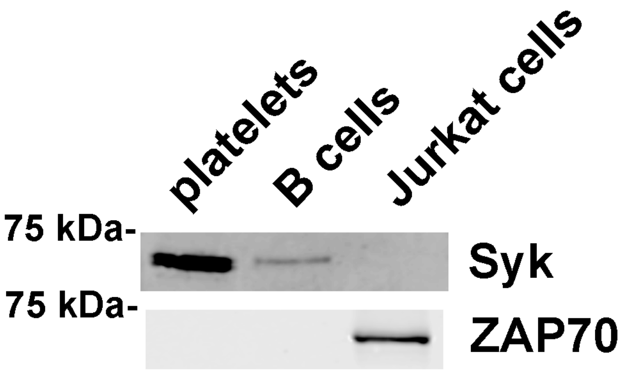

Figure S2

Platelets do not contain ZAP70.

Lysates from platelets, B cells or Jurkat cells were run on 8% SDS-PAGE, transferred to nitrocellulose and probed for either Syk or ZAP70.
