# Supplementary material for: Phosphorylation of spleen tyrosine kinase at Y346 negatively regulates ITAM-mediated signaling and function in platelets
Source: J Biol Chem. 2023 Jun 1;299(7):104865. doi: 10.1016/j.jbc.2023.104865 (PMC10320515; doi:10.1016/j.jbc.2023.104865)
Supplement: Supporting Figure S3 [file mmc3.pdf]

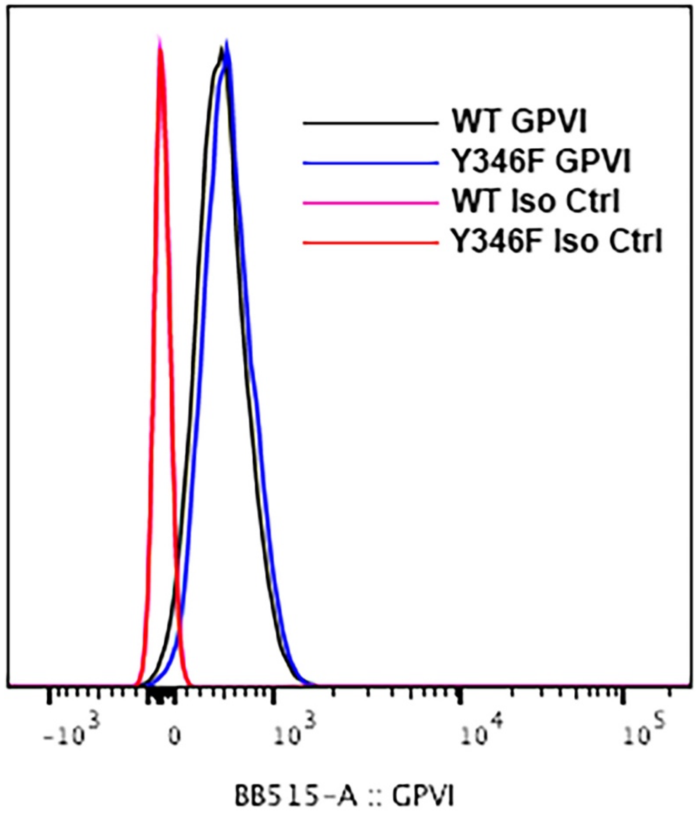

Figure S3.
Analysis of surface levels of GPVI by flow cytometry.
The surface level of GPVI was measured as described in the Methods.
